# Supplementary material for: A multiregional serosurvey of RNA virus-reactive antibodies in bats and rodents reveals the enzootic presence of flavi- and paramyxoviruses in African Pteropodidae
Source: PLoS Negl Trop Dis. 2026 Oct 9;20(10):e0013689. doi: 10.1371/journal.pntd.0013689 (PMC13674865; doi:10.1371/journal.pntd.0013689)
Supplement: Supplementary file 1 [file pntd.0013689.s015.pdf]

Response to the Editor:

1) We ask that a manuscript source file is provided at Revision. Please upload your manuscript file as a .doc, .docx, .rtf or .tex. If you are providing a .tex file, please upload it under the item type u2018LaTeX Source Fileu2019 and leave your .pdf version as the item type u2018Manuscriptu2019.

The revised manuscript will be uploaded as a .docx file.

2) Please insert an Ethics Statement at the beginning of your Methods section, under a subheading 'Ethics Statement'. It must include:
i) The full name(s) of the Institutional Review Board(s) or Ethics Committee(s)
ii) The approval number(s), or a statement that approval was granted by the named board(s).

The Ethics Statement, previously at the end of the document, has now been moved to the beginning of the Methods section. We apologize for the formatting oversight.

3) Please upload all main figures as separate Figure files in .tif or .eps format. For more information about how to convert and format your figure files please see our guidelines: https://journals.plos.org/plosntds/s/figures

All main figures will be uploaded in .tif format. We note that all main figures have undergone minor revision. The base layer to the map used in Figure 1 has been altered to ensure compliance with copyright rules. The map now originates from Natural Earth (https://www.naturalearthdata.com/), a public domain map database. Figure 2 has been revised to respond to Reviewer 2's suggestion of altering the size of the pie charts to reflect sampling effort. Figure 3 has been altered to reflect all samples included in this study, rather than only tropical bats.

Additionally, S4 Table has been added to report variance inflation factors (VIF) values for the logistic regression models to address concerns regarding multicollinearity.

4) Some material included in your submission may be copyrighted. According to PLOSu2019s copyright policy, authors who use figures or other material (e.g., graphics, clipart, maps) from another author or copyright holder must demonstrate or obtain permission to publish this material under the Creative Commons Attribution 4.0 International (CC BY 4.0) License used by PLOS journals. Please closely review the details of PLOSu2019s copyright requirements here: PLOS Licenses and Copyright. If you need to request permissions from a copyright holder, you may use PLOS's Copyright Content Permission form.

Please respond directly to this email and provide any known details concerning your material's license terms and permissions required for reuse, even if you have not yet obtained copyright permissions or are unsure of your material's copyright compatibility. Once you have responded and addressed all other outstanding technical requirements,

you may resubmit your manuscript within Editorial Manager. Potential Copyright Issues: i) Figure 1. Please (a) provide a direct link to the base layer of the map (i.e., the country or region border shape) and ensure this is also included in the figure legend; and (b) provide a link to the terms of use / license information for the base layer image or shapefile. We cannot publish proprietary or copyrighted maps (e.g. Google Maps, Mapquest) and the terms of use for your map base layer must be compatible with our CC BY 4.0 license. Note: if you created the map in a software program like R or ArcGIS, please locate and indicate the source of the basemap shapefile onto which data has been plotted. If your map was obtained from a copyrighted source please amend the figure so that the base map used is from an openly available source. Alternatively, please provide explicit written permission from the copyright holder granting you the right to publish the material under our CC BY 4.0 license.

We thank the editor for bringing this to our attention. As the basemap file was no longer available at a suitable resolution, a revised version of Figure 1 was created using a shapefile (ne_110m_admin_0_countries.shp) from Natural Earth (https://www.naturalearthdata.com/), a public domain map database.

Response to Reviewer 1:

The methods need to be explained in more detail in some areas:

-Line 187: which surgical procedures are the authors referring to in the frame of sampling? Drawing blood from a vein or artery would not be considered a surgical procedure, correct?

We thank the reviewer for this comment. Following application of anesthesia, some animals were necropsied at the time of blood/transudate sampling. We have now updated the text to reflect this.

Line 187: *"when surgical procedures were performed"* now replaced with Lines 230–232 (tracked changes version) in the revised version: *"prior to blood or transudate collection. Some animals were necropsied upon sampling, in which case blood was collected via cardiac puncture."*

Line 211: why were all OD values multiplied by 1,000? This step should be explained.

The figure actually reflects the raw OD values, not those multiplied by 1,000. We apologize for the oversight and the text has been updated accordingly.

Line 211: deleted *"and all optical density values were multiplied by 1,000."* (Line 254 in the revised version.)

Lines 234–248: why were STNs for YFV and MuV virus selected from all analyzed viruses? This should be explained for a better transparency.

YFV and MuV were selected as a prototypic flavi- and paramyxovirus, respectively, predominantly out of convenience due to prior experience working with these pathogens. As we had limited sample volumes available, the intention was merely to select a small subset of the examined viruses to determine whether mIIFT-positivity was an indicator of neutralizing activity.

Lines 234–248: added *"To determine whether mIIFT-positivity was an indicator of neutralizing activity, YFV and MuV were selected for neutralization tests based on prior experience of working with these pathogens (PMID: 22531181; PMID: 15705200)."* (Lines 279–281 in the revised version.)

Line 295: "if no information was available, a value of 12 years was assigned". How was that number selected? This needs to be explained.

We recognize that there is substantial uncertainty about the lifespan data used to inform our model. Therefore, we treat lifespan as an uncertain predictor, drawing the 'true' lifespan value from a log-normal distribution that is informed by the reported values in the literature. Combined with the selected value for the scale parameter, using 12 years to inform the location parameter for bat species with no lifespan information available, yielded a distribution with a lower quartile (Q1) of 6.7 years, an upper quartile (Q3) of 14.8 years, and a 5th–95th percentile range of 3.8 to 26.15 years, values consistent with typical average bat lifespans reported in the literature. Furthermore, lifespan data were unavailable for only two species: *Miniopterus inflatus* and *Coleura afra*.

Line 295 and the following sentences were significantly revised to Lines 345–353 in the revised version to explain our choice better: *"If no information was available, a value of 12 years was assigned as this fell within the range of known lifespan estimates (4.5–30 years) and approximates the mean lifespan estimate of all species included in this dataset. For Rhinolophus spp. obtained in Gabon, biological data from Rhinolophus landeri was used. For Hipposideros cf. caffer and Macronycteris gigas, we used the same lifespan expectancy as for Rhinolophus spp., as the corresponding families are closely related. Following manual exploration of implied log-normal distributions, using mean life expectancy values with varying degrees of spread, the following values for the scale parameter were selected:"*

The figures and tables are clear and of good quality.

Regarding the presentation of the results, I have the following suggestions:

Lines 367–373: I am not completely following this approach. If a bat species does not bind the anti-bat IgG conjugate equally well as others, but is still considered positive in the

mIIFT, how are the results evaluated then? A quantitative interpretation is precluded from this, but how is an unspecific reaction excluded? This whole approach needs to be explained in a more comprehensive matter.

The ELISA (S2 Fig.) was performed to evaluate whether the secondary antibodies (goat anti-bat IgG and goat anti-mouse IgG) used in the mIIFT could detect IgG originating from various bat and rodent hosts with comparable efficiency. Specific reactivity was observed for all species that were examined, indicating that the secondary antibodies could be used to detect all relevant host IgGs. The reviewer is correct to point out that secondary antibody binding affinity (as indicated by the ELISA results) varied by host, implying that mIIFT-sensitivity might also vary according to the host. In order to consider variable sensitivity of the secondary antibody binding, we applied a tertiary antibody (donkey-anti-goat-IgG Dylight488) to further amplify fluorescence signals in the mIIFT screening approach. To further confirm that samples from hosts exhibiting a low-degree of reactivity in the ELISA were still detectable by the mIIFT, samples from *Artibeus jamaicensis, Artibeus lituratus, Myotis dasycneme,* and *Nyctalus noctule* (species with relatively low ELISA-reactivity) were compared against *Rousettus aegyptiacus* (a species with high ELISA-reactivity). Even samples from species with low ELISA-reactivity exhibited apparently comparable fluorescent intensity as a sample from species with high ELISA reactivity (S3 Fig.), suggesting that putative host IgG-specific differences in assay sensitivity were not significant enough to preclude mIIFT readout.

Line 361–363: *"the reactivity of goat anti-bat and goat anti-mouse IgG conjugates was tested for each bat and rodent species used in this study"*

was replaced with

Lines 416–421 in the revised version *"an ELISA was performed to evaluate whether the secondary antibodies (goat anti-bat IgG and goat anti-mouse IgG) used in the mIIFT could principally detect IgG originating from various bat and rodent hosts with comparable efficiency. Although OD values varied among species, specific reactivity was observed for all species that were examined, indicating that the secondary antibodies could be used to detect all relevant host IgGs."*

Table 1: as mentioned above, I question the approach of comparing the seroprevalence in species that are only analyzed in one target region (e.g. Eidolon helvum and Rousettus aegyptiacus). For some other bat and rodent species (Epomops franqueti, Hypsignatus monstrosus, Micropterus puillus, Apodemus sylvaticus, Rattus norvegicus, Microtus arvalis, Clethrionomys glareolus) there are specimen from two countries available, but at low, sometimes simple-digit sample numbers. In my view, the validity of this approach needs at least a better explanation.

Line 389-390: “sera with high background signal or inconclusive results were rated negative”. Why were these samples not excluded from the study? Rating them negative will affect the overall result.

High background signal was generally apparent only in the absence of a positive signal, which exhibited relatively greater fluorescent intensity (see S3 Fig., S4 Fig.). Inconclusive results should be considered those which did not sufficiently meet the threshold for seropositivity. By rating these samples as negative we wanted to ensure that estimates for the seropositivity rates are conservative, limiting false positive detections as much as possible.

Lines 394–398: only one out of 20 tested mIIFT positive sera was reactive in the virus-specific SNT, which is here explained by a possible cross-reactivity with a related virus. The possibility of false positive mIIFT results needs to be discussed as well, and moved into the discussion section.

We thank the reviewer for this comment and we have amended the Discussion section accordingly:

Line 548 (Lines 618–621 in the revised version): we added *“Only 1/20 mIIFT-positive sera were reactive in virus-specific neutralization tests (Table 2), suggesting that the wild bats examined in this study had predominantly seroconverted against animal viruses, rather than directly against human pathogens.”*

Line 628 (Lines 703–712 in the revised version): we added *“To limit the possibility of false positive detection, the mIIFT was established using known positive human reference sera and cross-reactivity between viruses of the same group was established (Table S2). Plotting the Jaccard similarity of the samples gathered in this study reveals a similar pattern of cross-reactivity, i.e. among the orthoflaviviruses (DENV2, YFV, ZIKV), among the orthorubulaviruses (MuV, PIV2), and among the alphaviruses (CHIKV, VEEV) (Fig. 3) as in the human samples, suggestive of authentic mIIFT-reactivity specific to each virus genus. To a considerable extent, non-specific reactivity against host antigen can be excluded as a source of potential false positivity in the mIIFT assay, as only infected cells are fluorescent in the case of authentic detection.”*

I would also suggest to test the sera that are positive for YFV and DENV2 in a DENV2-specific neutralization assay.

We thank the reviewer for this suggestion. Unfortunately, the samples have already been used in several other studies (see e.g., PMID: 27217069; PMID: 25673701 (Eidolon); PMID: 24043818; PMID: 22696648; PMID: 22531181). Therefore, only residual sera were available for this study. There is insufficient volume to conduct additional neutralization assays, particularly as a low dilution (e.g., 1:20 in sample GH82) was required to effect YFV neutralization. As such, we cannot distinguish whether the flavivirus that gave rise to

this reactivity was more YFV- or DENV2-like. We note that a variety of African bat orthoflaviviruses (e.g., Bukalasa, Dakar, and Entebbe bat viruses) cluster together with an apparently similar antigenic profile (PMID: 6293325).

Line 401–404: again, how is a false positive result excluded here? > 45% of sera reacting with two or more virus families is a high number. How was this assured?

We cannot fully exclude the possibility that specific samples in our dataset may have exhibited non-specific reactivity. As mentioned in the introduction, we also did not aim to identify antibodies against specific pathogens with high diagnostic accuracy. However, to ensure high detection quality, the mIIFT specificity was established using known positive human reference sera (S2 Table) and positive detections among our samples exhibited an expected pattern of genus-specific cross-reactivity (Fig. 3) that is consistent with authentic detection.

Line 419–426: see above, not sure of analyzing data from species only samples in one zone are sufficiently relevant. For Rattus, samples from both zones were compared, but at relatively low numbers.

It is our view that in order to make a general comparison about whether seroprevalence is higher among bats and rodents living in tropical versus temperate regions, a more representative sampling strategy would be needed, and it cannot be determined whether differences in seroprevalence between tropical and temperate animals in our study is due to other confounding factors. The comparison among *Rattus* samples was performed, because as *Rattus* samples were available in both tropical and temperate regions, intrinsic host differences could be accounted for, but we agree with the reviewer that this provides insufficient support to infer a more general conclusion about the importance of climate zone. Our logistic regression analysis focused on tropical bats precisely because confounding factors among this subset of the data could be better controlled for, i.e., resulting in low VIFs for the independent variables in Model A (S4 Table).

Lines 458–460: "comparisons about differences in seroprevalence between bats and rodents or between tropical and temperate climate zones are not explicitly supported by our data", this means that the data argue against such a correlation? If so, this needs to be elaborated.

Please see the comment above. Although we observed higher rates of seroprevalence among tropical animals, given the limitations of the sampling strategy that was employed, we believe we have insufficient data to draw a strong conclusion about relative seroprevalence in tropical vs. temperate regions, particularly as many host-intrinsic factors were potential confounders. We specifically opted to perform logistic regression analysis on the tropical bat subset of the data for this reason.

Line 457–460: *“Due to sampling constraints and the presence of confounding factors, general comparisons about differences in seroprevalence between bats and rodents or between tropical and temperate climate zones are not explicitly supported by our dataset.”*

was replaced with

Lines 519–526 in the revised version: *“As described above, we detected higher rates of seropositivity in bats than in rodents and in animals living in tropical rather than temperate climate zones. As our samples were not representative of global populations, however, general comparisons about differences in seroprevalence between bats and rodents or between tropical and temperate climate zones are not explicitly supported by our dataset. We therefore limited our host characteristic analysis to the tropical bat subset of the data as described in the following section.”*

I have some general concerns about the interpretation of these data. The authors raise some limitations, but they do not offer a sound solution. This should in my view be discussed more transparently.

It is our intention to be as transparent as possible about the limitations of our dataset and modeling approaches. We hope that by appropriately qualifying our results, we convey clearly to the reader that we observe associations between host characteristics and seroprevalence, which are in some cases suggestive of possible routes of en- and epizootic transmission. Although such associations do not constitute definitive evidence for a particular mode of transmission, they can still offer valuable information to the reader, particularly when combined with other lines of evidence. For example, our modeling results were suggestive of bat-to-bat orthorubulavirus transmission among *R. aegyptiacus*, which is consistent with recent experimental findings about gastrointestinal shedding of orthorubulaviruses by *R. aegyptiacus* (PMID: 39770708 ref. 51,, see Lines 654-656 in revised version). We added: “*Orthorubulaviruses have been shown to shed from the gastrointestinal tract of R. aegyptiacus, suggesting that the fecal-oral route may be a common route of bat-to-bat orthorubulavirus infection [51].”*

My comments in detail:

Line 549–555: here, authors raise the points that in my view hamper the interpretation of the data in the way it is done here, but they don’t correlate these concerns with their interpretation, which nonetheless in both cases needs to be done.

Although we made every effort to include as many samples as possible in this work, our data are ultimately too sparse to separately evaluate all the variables that may be relevant to host seroprevalence.

Line 661 (Lines 745–749 in the revised version): we added *“Finally, we note that there are many host-extrinsic factors (e.g., seasonality, vector abundance) that may plausibly*

influence seropositivity, but were not considered by the present study. Future serosurveys may wish to consider sampling approaches that are explicitly designed to distinguish between host-intrinsic and -extrinsic risk factors."

Lines 572–580: the possibility of a fecal-oral flavivirus transmission in bats is valid and I agree that this should be further explored in relevant studies.

We thank the reviewer for this comment. The inference that the fecal-oral route seems to be a strong candidate for flavivirus transmission among bats is not something that we anticipated in advance of this study. Rather, the data we present (Fig. 2c, S6a Figure, S7a Figure) motivated this interpretation, and it seems to complement independent lines of evidence found in the literature (as discussed in Lines 648–656 in the revised version).

We have added in line 654–656 *"Orthorubulaviruses have been shown to shed from the gastrointestinal tract of R. aegyptiacus, suggesting that the fecal-oral route may be a common route of bat-to-bat orthorubulavirus infection (PMID: 39770708)."*

Lines 582–583: NiV is not a very good example for orthorubulavirus transmission.

We thank the reviewer for this comment. At the time the text was originally written, we were unaware of any studies explicitly looking at orthorubulavirus shedding in the bat mouth or gut, and therefore considered that orthorubulaviruses might spread between bats in a similar fashion to the henipaviruses (including NiV), for which oral shedding had already been experimentally demonstrated (PMID: 24626480). To some extent, the fact that our logistic regression model results were suggestive of a mode of transmission that has now been experimentally demonstrated lends them additional credibility.

Line 619: authors underline the high sensitivity of their approach, but how was the specificity (on virus family level) assured?

As stated above, the sensitivity of the mIIFT assay was originally established using human reference sera (Table S2).

Line 628 (Lines 703–712 in the revised version): we added *"To limit the possibility of false positive detection, the mIIFT was established using known positive human reference sera and cross-reactivity between viruses of the same group was established (Table S2). Plotting the Jaccard similarity of the samples gathered in this study reveals a similar pattern of cross-reactivity, i.e. among the orthoflaviviruses (DENV2, YFV, ZIKV), among the orthorubulaviruses (MuV, PIV2), and among the alphaviruses (CHIKV, VEEV) (Fig. 3) as in the human samples, suggestive of authentic mIIFT-reactivity specific to each virus genus."*

Line 633–634: the statement: "…makes this dataset unsuitable to broadly generalize about the relative seropositivity between …. tropical and temperate animals", but this is the message of this study. Please consider rephrasing to make it more clear.

Although we observed higher rates of seroprevalence among tropical animals, given the limitations of the sampling strategy that was employed, we believe we have insufficient data to draw a strong conclusion about relative seroprevalence in tropical vs. temperate regions, particularly as many host-intrinsic factors were potential confounders. We specifically opted to perform logistic regression analysis on the tropical bat subset of the data for this reason.

Line 457–460: *“Due to sampling constraints and the presence of confounding factors, general comparisons about differences in seroprevalence between bats and rodents or between tropical and temperate climate zones are not explicitly supported by our dataset.”*

was replaced with

Lines 519–526 in the revised version: *“As described above, we detected higher rates of seropositivity in bats than in rodents and in animals living in tropical rather than temperate climate zones. As our samples were not representative of global populations, however, general comparisons about differences in seroprevalence between bats and rodents or between tropical and temperate climate zones are not explicitly supported by our dataset. We therefore limited our host characteristic analysis to the tropical bat subset of the data as described in the following section.”*

Lines 638–652: when discussing the limitations, the authors should also suggest means to confirm their findings, i.e. follow-up analysis with virus specific tests, more SNTs etc., even if this was technically impossible due to the low residual sample volumes in many cases. Also, are there serum and transudate samples available from the same animals in this study to compare the validity of serum and transudate?

Unfortunately, only either blood or transudate was sampled for each individual animal, so we are unable to compare them directly. Transudates were only collected when blood collection was not feasible (e.g., in rodents with unusually small body size).

Line 652: the logistic regression models are extensively explained in the methods and results section, but only briefly discussed. It is concluded in the last paragraph that the findings only indicate correlations, but cannot prove them. In this light, the manuscript title as well as the overall message of this manuscript seem to over-interpret the findings.

We have amended the manuscript substantially in response to these concerns including changing the title (global was replaced by multiregional):

Lines 147–148: *“Our findings indicated a high seroprevalence among representatives of tropical bat genera, which prompted us to construct multivariable logistic regression models to evaluate tropical bat host characteristics as potential risk factors for infection.”*

was replaced with

Lines 152–163 in the revised version: *“Samples were obtained via a retrospective sampling strategy, which is a major limitation of this study. Although an effort was made to include as many samples as possible, samples in this study are not representative of global populations, and the data presented here are too sparse to make general conclusions about relative seroprevalence in bats versus rodents or in tropical versus temperate climate zones. Tropical bat samples comprised the bulk of our dataset (n=1,058/1,589), and originated from 12 distinct genera, reflecting diverse sets of host characteristics. Within our dataset, tropical bats exhibited especially high seroprevalence, prompting us to construct multivariable logistic regression models to evaluate tropical bat host characteristics as potential risk factors for infection.”*

Line 661 (Lines 745–749 in the revised version): we added *“Finally, we note that there are many host-extrinsic factors (e.g., seasonality, vector abundance) that may plausibly influence seropositivity, but were not considered by the present study. Future serosurveys may wish to consider sampling approaches that are explicitly designed to distinguish between host-intrinsic and -extrinsic risk factors.”*

It was our aim with this manuscript to provide an overview of seroprevalence against diverse RNA viruses with spillover potential in bats and rodents. Rather than merely report our results in terms of genus-specific seroprevalence (as in Fig. 2), logistic regression was used to disambiguate tropical bat host characteristics to identify host-intrinsic correlates of seropositivity. We do not assert that our data are sufficient to establish a causative link, but they rather indicate associations which are suggestive of plausible routes of transmission.

We believe that the data we provide are sufficient to justify the assertion that paramyxo- and flaviviruses are enzootic in African Pteropodidae (see Lines 502–509 in the revised version). To allay concerns about false positive detection, additional reasoning has also been provided as previously mentioned above. See Lines 703–712 in the revised version: “*To limit the possibility of false positive detection, the mIIFT was established using known positive human reference sera and cross-reactivity between viruses of the same group was established (S2 Table). Plotting the Jaccard similarity of the tropical bat samples gathered in this study reveals a similar pattern of cross-reactivity, i.e. among the orthoflaviviruses (DENV2, YFV, ZIKV), among the orthorubulaviruses (MuV, PIV2), and among the alphaviruses (CHIKV, VEEV) (Fig. 3), suggestive of authentic mIIFT-reactivity specific to each virus genus. To a considerable extent, non-specific reactivity against host antigen can be excluded as a source of potential false positivity in the mIIFT assay, as only infected cells are fluorescent in the case of authentic detection.”*

This manuscript follows a serological approach to assess the prevalence of RNA viruses in bat and rodent species in different regions of the world. The multiplex approach by using the mIIFT is very suitable to address this. Although the scientific question behind the study is highly relevant, in my view the available data do not allow all conclusions drawn from it.

My main concern is the comparability between frugivorous bats in tropical regions, and insectivorous bats in the Northern hemisphere. These are two fundamentally different scenarios with a whole number of variabilities, that I find it hard to point all observed differences to the bat or rodent species. In my view this is a considerable weakness of the study, and this point needs to be carefully addressed and discussed.

We agree with the reviewer that it would be wrong to conclude from the present work that animals in tropical climate zones have higher rates of seropositivity than animals in temperate climate zones. Nor should one conclude from the present work that bats have higher rates of seropositivity than rodents. We think our dataset is insufficiently representative of such broad populations to make such claims. Nevertheless, within our dataset, we observed a tendency for tropical bats to have the highest rates of seropositivity and offer this observation as a factual description of our study population.

Regarding diet, we focused specifically on the tropical bat subset of the data because we had a sufficient sample size and diversity of host genera to consider a modeling approach that might help us distinguish between potentially relevant risk factors. The tropical bat samples (n=1,058) in this study spanned 12 distinct genera, including both frugivorous and insectivorous species of Yango- and Yinpterochiroptera. The low VIFs (S4 Table) in Model A emphasize the degree to which host characteristics could be treated as independent variables, given the diversity of genera that were sampled. Our conclusions in the discussion regarding the importance of frugivory are based on the logistic regression analyses we performed (S6 Fig. and S7 Fig.) solely on the basis of tropical bat data.

As explained in the comments above, we have significantly amended the manuscript to state our position more clearly.

My comments in detail:

The title mentions a global serosurvey. In fact, samples from seven countries on four continents were analyzed, which is not a global survey.

We thank both reviewers for raising this point and agree that “multiregional” is a more fitting descriptor than “global.” We have updated the title accordingly. It was our intent to highlight that our sampling efforts were not restricted to any single geographic region and we agree that our samples are not representative of global populations.

Author summary:

Line 82/83: “we studied bats and rodents around the world” should be worded more carefully

Corrected to “at 63 sites in seven countries” (Line 85 in the revised version).

Line 83: 14 viruses, add here: from 7 virus families

Corrected. (now line 86)

Introduction

Line 100: mention the goal / the benefit such studies would have.

We address this point in the following two places in the Introduction:

Line 115–117 in the revised version: "*Better identification of virus hotspots in nature may lead to targeted measures to reduce the risk of zoonotic spillover and contribute to "primary pandemic prevention (PMID: 35119921)*"

Line 129–132 in the revised version: *"A better understanding of infection rates in bats and rodents might help discern between enzootic and epizootic hosts and reveal unknown maintenance cycles amenable to control measures.*"

Lines 108–111: the limitations referred to in general (low number of analyzed virus families / low number of sampled species per country) are also somewhat limiting the present study. This point should therefore be raised also in the discussion.

To our knowledge, this is the most extensive serosurvey ever performed on tropical bats in terms of both virus and host diversity using a comparable methodology. Many serological surveys restrict themselves to either a single host species or a single pathogen. We agree with the reviewer, however, that it is important to be mindful of sampling limitations when considering whether tendencies observed with study populations are reflective of more global trends.

Lines 125–127: the authors then suggest a "systematic investigation of infection rates in bats and rodents", which in my view needs to include sampling the same bat or rodent species at relevant numbers in different global regions, which was not pursued in the present study.

Replaced with *"a better understanding of infection rates in bats and rodents."* Whereas many serological studies focus on a single species or single pathogen, we wanted to use a comparable methodology to examine numerous species and pathogens for which samples were readily available. Our aim was to maximize the number of samples included in the dataset, but we acknowledge that the sampling strategy is a major limitation of the study design.

Line 141: exposure to filovirus antigen (instead of 'with')

Corrected.

Response to Reviewer 2:

This manuscript presents serological screening of bats and rodents across seven countries for antibodies to 14 human pathogenic RNA viruses. While the study addresses an important question about wildlife reservoir dynamics, several fundamental issues with study design, geographic scope, and analytical approach limit the validity and generalisability of the findings.

We agree with the reviewer that the study design, especially the convenient sampling approach, imposes a limitation on what general conclusions may be drawn from the present work. Still, we are convinced that the data set is highly valuable and is probably one of the largest comparative serology studies done based on a comparative method. Our aim is to make our data publicly available and to be transparent about the representativeness of our sampling approach. Importantly, we have made substantial revisions to the text of the manuscript to better distinguish when we are describing observations of our study population versus any general conclusions with regard to risk factors. It is our view that our analyses offer useful indications about potential modes of orthoflavi- and orthorubulavirus transmission among Pteropodidae bats, despite the mentioned limitations. We address all the specific points raised below.

Major issues

-The use of "global" to describe this study is inappropriate and misleading. Seven countries, comprising two in Western Europe, three in West/Central Africa, one in SE Asia and one in Central America does not constitute global coverage. Major geographic regions including North America, South America, Australasia, and vast portions of Asia are completely unrepresented. The current terminology misrepresents the scope of the work and risks readers drawing conclusions about worldwide patterns that are not supported by the data. This is especially the case given that only 1% of bat species and 0.5% of rodent species are sampled, and sampling is highly biased - 1015/1135 (89%) of bats were sampled in West/Central Africa, with the remaining in Germany (n=77 bats) and Panama (n=43 bats), and none in other sites. By comparison, rodents are sampled in Western Europe (n=115 in Germany and n=165 in the Netherlands) and Thailand (n-174), with none in Africa or the Americas. My concern with 'global' is easily fixed - it only appears in the title. Authors should remove "global" and frame alternatively as "multi-country" or "intercontinental".

We thank both reviewers for raising this point and agree that “multiregional” is a more fitting descriptor than “global.” We have updated the title accordingly. It was merely our intent to highlight that our sampling efforts were not restricted to any single geographic region. We agree that our samples are not representative of global populations.

However, the generalisation of results from a small and biased sample set is more prevalent throughout - it is inappropriate to generalise findings within the abstract as applying to "tropical bats" when using such a narrow sample set, or by variables such as

colony size, when there are so many potential confounders and too small a sample size to tease these confounders apart. The abstract and manuscript need a major reframing to avoid overstating the results.

We recognize that the sampling is not evenly distributed among the regions that were included in this study, a feature that we characterize as a substantial limitation.

For example, in Lines 715–721 in the revised version, we state: *“Moreover, as samples were included in this study based on their availability, rather than a pre-meditated sampling strategy, confounding factors make this dataset unsuitable to broadly generalize about the relative seropositivity between bats and rodents or tropical and temperate animals. For example, no African rodents were included as a part of this study, and no temperate regions were included outside of Europe. Future serosurveys should employ sampling strategies which ensure that target species better reflect geographic diversity within each taxonomic order.”*

To further qualify our results, we have made additional amendments to the Abstract, Author Summary, and Discussion, including statements which better clarify the limitations of our study (e.g., in Lines 72–73 in the revised version, we now state: *“confounding factors and sparse data continue to pose challenges to identifying risk factors for RNA virus infection in wildlife”*).

Nevertheless, it is our view that the data we provide are sufficiently informative to be of some practical use despite the limitations of our sampling strategy. Most serosurveys restrict themselves to a single species, a single pathogen, and/or a single region. Our aim in this work was to use all samples that were available to us, so that our modeling efforts could be informed by as much data as possible. We recognize that the models presented in this study are provisional, and expect them to be further refined as more data becomes available. If we were to have restricted ourselves to a purely descriptive approach (e.g., presenting the data as in Figure 2 without further analysis), there would also be a danger of giving the impression that risk of infection depends primarily on genus identity, without addressing underlying factors. One of the advantages of implementing a modeling approach is that it highlights a complex array of interdependent variables which may contribute to seropositivity, and thereby provides a principled basis upon which to begin evaluating their relative importance. By providing a set of potentially relevant host characteristics and initial data, our results may help inform future study design, including studies explicitly aimed at discriminating among potential confounders.

Additionally, results like Figure 2 must take into account sampling effort. It is meaningless to compare the number of viruses detected in genera from which one rodent (Microtus) or 14 individual bats (Coleura) were collected versus those where nearly 200 individual bats were sampled (Rousettus). A genus with one seropositive individual will show 100% of one viral family, while a genus with 200 sampled individuals has far more opportunity to

detect multiple families. Analyses must account for differences in sample size before making such comparisons, or the figure adjusted so that the size of the circles represents sample size.

We appreciate the reviewer´s comment and fully agree that it would be more informative to visualize the varying sample sizes between the different genera in Figure 2. A revised version of Figure 2 now explicitly reflects differences in sample size.

New Figure 2:

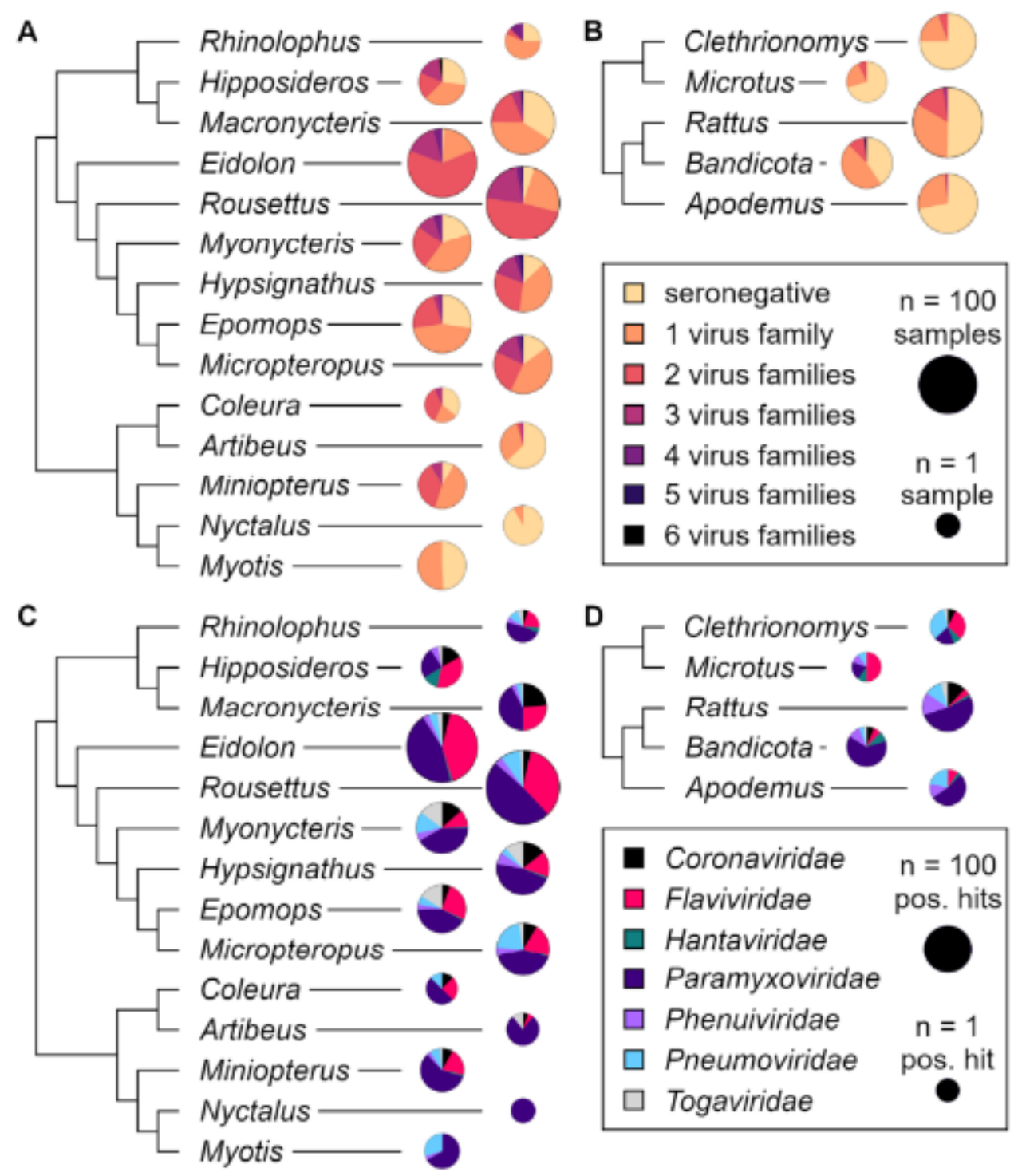

-Seasonality, reproductive status or age of individuals sampled is not addressed in the methods, despite it being well documented as a determinant of serostatus and included in the modelling later on. The effect of these covariates have also been shown to vary by viral family and host species. The absence of recognition of this within the study design, manuscript, and provision of data is a major gap.

We would like to emphasize that seasonality is addressed in a later comment (see below). The sex and age of individuals was included in our logistic regression analysis and is reported (S3 table). As all samples derived from wild animals, precise ages were not known, but were reported as either adult, subadult, or juvenile.

-The choice of viruses included in the panels are not sufficiently justified. I can understand why most of the human viruses were included, but the rationale for excluding viral genera that are known to have strong associations with the bat and rodent reservoirs being screened is unclear (e.g. the huge diversity of paramyxoviruses that this group and others

have demonstrated in African bats - but are only distantly related to measles and mumps). This includes viruses (or very close relatives of viruses) that are known to have transmitted to humans and for which control sera would have been available (e.g. pararubulavviruses and henipaviruses).

We thank the reviewer for this observation. Our intent was to select a panel of known human RNA virus pathogens such that we could detect related (potentially zoonotic) viruses circulating among bats. We recognize that there are notable zoonotic RNA virus groups found in bat and/or rodent reservoirs that remain absent from our panel (e.g., filoviruses, henipaviruses, arenaviruses). As we used whole virus antigen in the mIIFT, we were dependent upon the availability of characterized virus stocks and the inclusion of biosafety level 4 viruses was precluded due to logistical constraints by the company EUROIMMUN.

-The methods do not describe how seropositivity was determined for each assay (I see it is later presented in the results instead) and the reason for the use of VNTs for YFV was not described.

-The abstract implies that tropical vs temperate bats were statistically compared, but this is not the case.

In absolute terms, we observed a higher rate of seropositivity among the tropical versus the temperate samples within our dataset, but we explicitly state that our data do not support a general comparison between these groups due to confounding factors. We have amended the text to better clarify our position:

Lines 151–161 in the revised version: *"Samples were obtained via a retrospective sampling strategy, which is a major limitation of this study. Although an effort was made to include as many samples as possible, samples in this study are not representative of global populations, and the data presented here are too sparse to make general conclusions about relative seroprevalence in bats versus rodents or in tropical versus temperate climate zones. Tropical bat samples comprised the bulk of our dataset (n=1,058/1,589), and originated from 12 distinct genera, reflecting diverse sets of host characteristics. Within our dataset, tropical bats exhibited especially high seroprevalence, prompting us to construct multivariable logistic regression models to evaluate tropical bat host characteristics as potential risk factors for infection."*

Lines 519–526 in the revised version: *"As described above, we detected higher rates of seropositivity in bats than in rodents and in animals living in tropical rather than temperate climate zones. As our samples were not representative of global populations, however, general comparisons about differences in seroprevalence between bats and rodents or between tropical and temperate climate zones are not explicitly supported by our dataset. We therefore limited our host characteristic analysis to the tropical bat subset of the data as described in the following section."*

Similarly, it is not clear in the abstract that no statistical analyses were performed on rodent results. The authors restrict analyses to the tropical bat data for further analyses because it has high seroprevalence, removing the opportunity of comparing with low-prevalence bats. Using regional differences in sample size as an argument for this approach serves to highlight the weakness of the study design in the first place. The authors admit that "many bat families were only sampled in one region" and that collinearity and confounding are substantial problems, but proceed with the subsetted analyses anyway. Saying "This should be borne in mind when interpreting" is insufficient when the confounding is this severe.

We acknowledge that the retrospective sampling strategy used in this study is a substantial limitation. However, instead of merely providing a descriptive summary of the entire dataset, we opted to implement two modeling approaches on the tropical bat subset of the data in order to gain some first insight into whether specific features of tropical bat genera were associated with seropositivity against three virus families. This particular subset of the data was selected due to large sample size, high rates of seropositivity, and low multicollinearity when a subset of relevant host traits were considered. Model A was explicitly designed to address concerns regarding multicollinearity (resulting in VIFs < 5, see newly-added S4 Table) and Model B was designed to incorporate all sample variables for which data was available.

Some additional caveats have been added throughout the manuscript to avoid overstating our findings. For example:

Line 72–73 in the revised version: Although confounding factors and sparse data continue to pose challenges to identifying risk factors for RNA virus infection in wildlife, multivariable logistic regression models were created to provisionally evaluate the association of tropical bat characteristics with seropositivity.

Our intention is to be transparent about the limitations of our study, so that future models, predicated upon more data, may refine or replace the framework we provide.

Lifespan data are notoriously unreliable and inconsistently determined. Here, where there is missing data for a particular species the authors borrow data from related species, introducing an additional layer of uncertainty. Then, arbitrary assignment of 12 years when no data exist also introduces substantial uncertainty - all of which propagate through the analyses (leading to convergence issues later on).

Line 295 and the following sentences were significantly altered to address this remark.

Lines 345–353 in the revised version: *"If no information was available, a value of 12 years was assigned as this fell within the range of known lifespan estimates (4.5–30 years) and approximates the mean lifespan estimate of all species included in this dataset. For Rhinolophus spp. obtained in Gabon, biological data from Rhinolophus landeri was used. For Hipposideros cf. caffer and Macronycteris gigas, we used the same lifespan*

expectancy as for Rhinolophus spp., as the corresponding families are closely related. Following manual exploration of implied log-normal distributions, using mean life expectancy values with varying degrees of spread, the following values for the scale parameter were selected:"

-I have not commented in detail on the results or discussion, as I feel the issues highlighted above with the methods (e.g. providing sufficient detail/raw data on the age and reproductive status of individuals sampled and the seasonality of sampling) preclude interpretation. The statistical models show substantial uncertainty (wide credible intervals) that undermine the conclusions about risk factors

We recognize that there is substantial uncertainty about the lifespan data used to inform our model. Therefore, we treat lifespan as an uncertain predictor, drawing the 'true' lifespan value from a log-normal distribution that is informed by the reported values in the literature. Combined with the selected value for the scale parameter, using 12 years to inform the location parameter for bat species with no lifespan information available, yielded a distribution with a lower quartile (Q1) of 6.7 years, an upper quartile (Q3) of 14.8 years, and a 5th–95th percentile range of 3.8 to 26.15 years, values consistent with typical average bat lifespans reported in the literature (see GitHub link for details). We do recognize that this adds uncertainty to parameter estimates, reflecting the actual uncertainty in the data.

Regarding seasonality, all sampling dates are also provided in Table S3. Seasonality alone is a difficult variable to treat appropriately in the context of a multiregional study, where seasonal dynamics vary significantly between regions or even among sites within the same region. In addition to seasonality, a variety of other extrinsic factors, including climate- and ecology-related variables, could plausibly play a role in seropositivity. Sampling year- and region-specific differences in seropositivity rate would be expected to appear in the "year_region" parameter (S7 Fig.). Instead of focusing on these extrinsic factors, however, the main focus of the paper was to identify intrinsic (host-related) risk factors, as these were regarded as potentially being more informative about putative routes of transmission. Ultimately our data appear too sparse to draw any strong conclusions about extrinsic factors. To address the reviewer's concerns, we have added an explicit statement to the Discussion:

Line 661 (Lines 745–749 in the revised version): we added *"Finally, we note that there are many host-extrinsic factors (e.g., seasonality, vector abundance) that may plausibly influence seropositivity, but were not considered by the present study. Future serosurveys may wish to consider sampling approaches that are explicitly designed to distinguish between host-intrinsic and -extrinsic risk factors."*

We agree with the reviewer that wide credible intervals are indicative of model uncertainty. We think this is partly due to the sparsity of seropositive samples. Nevertheless, large colony size was positively associated with orthorubula- and orthoflavivirus seropositivity

both in model A and model B, such that 0 was not in range of the credible interval. This suggests that colony size can exert an important influence on seropositivity. While we agree that more data would be needed to make a definitive assertion colony size plays a causative role in high seroprevalence, our analysis lends some degree of support to the hypothesis that critical community size may be an important factor for the maintenance of paramyxoviruses in bat populations (PMID: 24253424).

-The github link was broken

We apologize for the oversight. The Github repository is now publicly accessible here: https://github.com/VirologyCharite/miift-serosurvey
