# Supplementary material for: A multiregional serosurvey of RNA virus-reactive antibodies in bats and rodents reveals the enzootic presence of flavi- and paramyxoviruses in African Pteropodidae
Source: PLoS Negl Trop Dis. 2026 Oct 9;20(10):e0013689. doi: 10.1371/journal.pntd.0013689 (PMC13674865; doi:10.1371/journal.pntd.0013689)
Supplement: Supplementary file 3 [file pntd.0013689.s013.docx]

**S7 Fig.**

**A**

**B**

**C**

**D**

**E**

**S7 Fig. Parameter sizes estimated by multivariable logistic regression using model B.** Posterior distribution of estimated sizes for regression parameters, given as expected change in log odds of detecting seropositivity for 11 viruses found in tropical bats. Horizontal black bars beneath distributions indicate the 94% (thin bar) and 50% (thicker and shorter overlapping bar) highest posterior density intervals. Estimated associations of the following sample characteristics with seropositivity are shown: membership in the taxonomic suborder Yangochiroptera (as opposed to Yinpterochiroptera), migratory lifestyle (as opposed to sedentary lifestyle), insectivorous diet (as opposed to frugivorous diet), cave-dwelling (as opposed to tree-dwelling), Log(colony size) (shown for 5 units on the natural log scale), adult (as opposed to juvenile), male (as opposed to female), bat family, and year_region, a variable comprising both the region and year of sampling. Results shown for **(a)** flaviviruses, **(b)** paramyxoviruses, **(c)** togaviruses, **(d)** coronaviruses, and **(e)** a phenuivirus.
