# Supplementary material for: A multiregional serosurvey of RNA virus-reactive antibodies in bats and rodents reveals the enzootic presence of flavi- and paramyxoviruses in African Pteropodidae
Source: PLoS Negl Trop Dis. 2026 Oct 9;20(10):e0013689. doi: 10.1371/journal.pntd.0013689 (PMC13674865; doi:10.1371/journal.pntd.0013689)
Supplement: Supplementary file 4 [file pntd.0013689.s016.docx]

We thank the reviewers and editors for their constructive comments, which have helped us to improve the clarity and rigor of the manuscript. Please find our point-by-point reply below marked in blue.

**Reviewers' Comments:**

**Summary and General Comments**

**Reviewer #3**: I would like to start by emphasizing that I find this manuscript valuable and scientifically interesting. The study is based on an impressive and unusually diverse sample set, including 1,135 bat and 454 rodent specimens collected from 63 sites across seven countries, and screened against 14 RNA viruses from seven virus families. I particularly appreciate the scale of the work and the effort required to assemble and analyse material from multiple host species, countries, and ecological settings. The multiplex immunofluorescence approach is well suited for such a broad exploratory serosurvey, especially given the known limitations of direct virus detection in field-collected wildlife samples. Overall, I consider this a useful contribution to wildlife virus surveillance. My comments below are intended mainly to help strengthen the interpretation of the data, particularly regarding the statistical analyses and the generalizability of the conclusions.

1. The statistical analysis is generally well described and represents a reasonable exploratory attempt to extract information from a heterogeneous retrospective dataset. It is more careful than the typical wildlife serosurvey regression, and the authors have clearly already tightened their causal language substantially in response to concerns about overgeneralization. However, the models should still be interpreted strictly as hypothesis-generating. Because many predictors are inferred at the species level while serostatus is measured at the individual level, and because species, family, geography, year, diet, colony size, and roosting ecology are strongly confounded, the models cannot robustly identify independent causal risk factors. I therefore recommend replacing “risk factor” throughout the abstract and discussion with more cautious wording, such as “associated factor”, “correlate”, or “predictor within this dataset”.

**REPLY**: We thank the reviewer for this comment and agree that the models should be interpreted as hypothesis-generating. They were intended to identify correlates of seropositivity, and we agree that no causal inference can be made solely on the basis of our dataset. We have adapted the language of the text to better reflect this view, and have replaced “risk factor” throughout the text where it referred to our results. For clarification, our view is that certain patterns were suggestive of highly plausible modes of transmission and are worthwhile identifying for the reader as potential lines of future investigation. We also note that Model A was specifically designed to limit multicollinearity, resulting in low variance inflation factors (VIFs). Although Model A had the advantage of limiting the degree to which the selected variables confounded each other, it had the disadvantage that potentially determinative underlying variables (e.g., species, sampling site) were omitted.

2. The presentation of the modelling results would benefit from a concise main-text table summarizing the posterior estimates and 94% credible intervals for the main predictors that are discussed biologically, especially colony size, frugivory/cave-dwelling, family, and region/year effects. At present, some important statistical evidence is buried in the supplementary material. Such a table would make it easier for non-statistical readers to assess which conclusions are well supported and which remain highly uncertain.

**REPLY**: We have added a table to the main text (Table 3) and to the supplement (new Table S4), which report 94% credible intervals for models A and B, respectively. The full table, including mean, standard deviation, and r_hat values is still available in the Supplementary Material on the GitHub repo: <https://github.com/VirologyCharite/miift-serosurvey>

| **Table 3. HPDIs for logistic regression model A.** | | | | | | | | | | | | | | |
| --- | --- | --- | --- | --- | --- | --- | --- | --- | --- | --- | --- | --- | --- | --- |
| **parameter** | **DENV-2** | | **YFV** | | **WNV** | | **MeV** | | **MuV** | | **PIV-1** | | **PIV-2** | |
|  | **hdi_3%** | **hdi_97%** | **hdi_3%** | **hdi_97%** | **hdi_3%** | **hdi_97%** | **hdi_3%** | **hdi_97%** | **hdi_3%** | **hdi_97%** | **hdi_3%** | **hdi_97%** | **hdi_3%** | **hdi_97%** |
| Intercept | -3.86 | -2.42 | -3.53 | -2.14 | -4.68 | -2.98 | -4.02 | -2.48 | -2.47 | -1.32 | -3.08 | -1.82 | -2.82 | -1.6 |
| Yangochiroptera | -1.35 | -0.11 | -1.37 | -0.15 | -1.17 | 0.24 | 0.16 | 1.18 | -0.77 | 0.21 | 0.08 | 1.03 | -0.61 | 0.42 |
| Migratory | -0.16 | 1.04 | -0.18 | 1 | -0.4 | 0.98 | -0.21 | 0.91 | 0.11 | 1.09 | 0.21 | 1.29 | 0.15 | 1.21 |
| Insectivorous | -1.59 | -0.68 | -1.63 | -0.71 | -2.15 | -1.07 | -0.27 | 0.78 | -1.89 | -1.05 | -1.2 | -0.29 | -2.61 | -1.65 |
| Cave-dwelling | 0.42 | 1.17 | 0.17 | 0.9 | 0.33 | 1.11 | -0.16 | 0.84 | 0.56 | 1.26 | -0.6 | 0.1 | 0.62 | 1.49 |
| Ln(colony size) (per 5 log units) | 1.34 | 1.75 | 1.27 | 1.66 | 1.39 | 1.84 | -0.78 | -0.04 | 0.65 | 0.98 | 0.02 | 0.34 | 1.03 | 1.44 |
| Adult | 0.06 | 0.76 | 0 | 0.69 | -0.09 | 0.68 | -0.04 | 0.97 | 0 | 0.62 | 0.13 | 0.84 | 0.06 | 0.74 |
| Male | -0.19 | 0.43 | -0.21 | 0.38 | -0.23 | 0.45 | 0.02 | 0.84 | -0.32 | 0.21 | -0.18 | 0.39 | -0.34 | 0.24 |
|  |  |  |  |  |  |  |  |  |  |  |  |  |  |  |
| **parameter** | **CHIKV** | | **VEEV** | | **hCoV-229E** | | **SARS-CoV** | | **RVFV** | | **RSV** | |  |  |
|  | **hdi_3%** | **hdi_97%** | **hdi_3%** | **hdi_97%** | **hdi_3%** | **hdi_97%** | **hdi_3%** | **hdi_97%** | **hdi_3%** | **hdi_97%** | **hdi_3%** | **hdi_97%** |  |  |
| Intercept | -3.22 | -1.46 | -3.68 | -1.73 | -2.69 | -1.23 | -3.4 | -1.88 | -3.55 | -1.86 | -3.42 | -1.89 |  |  |
| Yangochiroptera | -1.31 | 0.14 | -1.02 | 0.53 | -1.17 | 0.11 | -0.9 | 0.32 | -1.24 | 0.17 | -0.75 | 0.48 |  |  |
| Migratory | -0.89 | 0.48 | -0.51 | 0.98 | -0.92 | 0.21 | -0.1 | 1.13 | -0.67 | 0.65 | 0.05 | 1.32 |  |  |
| Insectivorous | -1.16 | 0.24 | -1.19 | 0.35 | 0.44 | 1.57 | -0.07 | 1.01 | -0.95 | 0.27 | -1.3 | -0.27 |  |  |
| Cave-dwelling | -1.47 | -0.28 | -1.28 | 0.22 | -0.62 | 0.56 | -0.78 | 0.21 | -0.56 | 0.45 | -0.18 | 0.63 |  |  |
| Ln(colony size) (per 5 log units) | -0.85 | -0.19 | -2.47 | -0.9 | -0.98 | -0.06 | -0.58 | -0.01 | -0.43 | 0.18 | -0.58 | -0.08 |  |  |
| Adult | -0.06 | 1.05 | -0.13 | 1.07 | -0.83 | 0.11 | -0.37 | 0.52 | -0.34 | 0.68 | -0.03 | 0.82 |  |  |
| Male | -0.1 | 0.79 | 0.04 | 1.01 | -0.36 | 0.53 | -0.42 | 0.37 | -0.34 | 0.54 | -0.2 | 0.49 |  |  |

**Reviewer #4**: The present manuscript by J Emanuel et al. reported comprehensive serological surveillance of bats captured in various regions in the world. Since the study design mostly rely on immunofluorescence assays using virus-infected cells as antigens and only partly incorporate neutralization tests to validate the results, as the authors are aware, the present study must contain several limitations to interpret. However, the attempts to draw a broad overview of the serological presence of viral infections in bats, which are considered as the most important group of animals in the context of emerging infectious diseases, should be valuable for sharing with researchers in this field. I would like to raise some issues of the present manuscript below for improving the manuscript before further consideration, while I am not 100% sure that these comments are not against previous suggestions of reviewers.

1) The present study used 14 human pathogens as antigens. While the authors confirmed the reactivity of antisera for 17 pathogens to evaluate the assays, this reviewer has not been satisfied with this evaluation. Since the cross-reactivity can largely be explained by the amino-acid sequence identity between antigens, especially major antigens, such as nucleoproteins, matrix proteins, and envelope proteins, the authors may be able to show the potential coverage of their serological assays. In example, the cross-reactivity among flaviviruses may be dependent on the genetic complexes (doi: 10.3389/fimmu.2020.00334). The authors may be able to speculate the cross-reactivity using sequence comparison as well as literature review. Visualization of the coverages by either a figure or a table may be helpful for readers to understand how the results can be explained. Furthermore, I’m interested in whether bat viruses or bat-origin emerging viruses are included or not in the potentially cross-reactive viruses.

**REPLY**: We thank the reviewer for raising this point. A key distinction should be made between co-reactivity and cross-reactivity (e.g., between virus A and virus B). To clarify, without knowing the complete infection history of the host, we can only directly measure co-reactivity, which effectively acts as an upper bound for cross-reactivity. Co-reactivity may either be due to infection by a single virus, which results in reactivity against both virus A and virus B (i.e. authentic cross-reactivity) or co-reactivity may be due to two or more independent infections (i.e. exposure to two or more distinct sets of antigens) which result in observed reactivity against both virus A and virus B. At several points in the text (e.g., lines 387, 388, 393, 603, 739) we now refer to co-reactivity instead of cross-reactivity where appropriate.

Detection of cross-reactivity depends heavily upon the target antigen and the assay method that is used. Previous reports have found substantial within-genus cross-reactivity among the orthoflaviviruses and orthorubulaviruses (PMID: 32174923; PMID: 2545038). In our case, we used whole virus antigen and the mIIFT, which we expect to have resulted in broad cross-reactivity within genera. It is also plausible that a lesser degree of cross-reactivity might occur across genera within the same virus family.

To specifically address the reviewer’s point, we have now calculated pairwise odds ratio values for all 14 viruses examined in this study (S6 Table). High odds ratio values (6.85–121) for seropositivity against viruses of the same genus are direct evidence of co-reactivity and suggestive of within-genus cross-reactivity. To determine whether there was a correlation between the odds ratio of seropositivity and amino acid identity of the expected gene products, a plot was made (now available in the GitHub repo as an interactive .html file and here below: <https://github.com/VirologyCharite/miift-serosurvey/results/identity_vs_odds_ratio.html>). The pairwise amino acid identity (following MAFFT alignment) of seven known bat flavi- and paramyxoviruses (Achimota virus 1, NC_025403.1; Achimota virus 2, NC_025404.1; bat paramyxovirus AR1, NC_038271.1; Bukalasa bat virus, OL709419.1; Dakar bat virus, OL709418.1; Entebbe bat virus, NC_008718.1; Ghana virus, NC_025256.1) was evaluated with regard to the 14 viruses included in this study. The bat viruses fell within a range (32.8–76.3% pairwise aa identity to the closest matching of the 14 viruses that were tested) such that cross-reactivity might be expected.

We have altered the text in the Results section (Lines 607–619) to read:

*“To further evaluate potential cross-reactivity among all sera, the odds ratios were considered, and Fisher P and Benjamini-Hochberg Q-values were calculated (S6 Table). As expected, high odds ratio values for seropositivity against viruses of the same genus (6.85-121) are suggestive of within-genus cross-reactivity. To ascertain to what degree bat viruses would also be expected to exhibit cross-reactivity with the viruses examined in this study based on their gene products, seven known bat viruses (Achimota virus 1, NC_025403.1; Achimota virus 2, NC_025404.1; bat paramyxovirus AR1, NC_038271.1; Bukalasa bat virus, OL709419.1; Dakar bat virus, OL709418.1; Entebbe bat virus, NC_008718.1; Ghana virus, NC_025256.1) were selected and pairwise amino acid identities of all MAFFT-aligned sequences were calculated. Reference sequences used for amino acid identity calculation and a visualization of the results are available at: <https://github.com/VirologyCharite/miift-serosurvey>.”*

We also note that due to the relatively high sequence homology between viruses of distinct, but closely related genera (e.g., between the pararubula- and orthorubulaviruses), we cannot exclude that cross-reactivity across genera within the same virus family is also occurring. For this reason, we have replaced “orthorubulavirus” with “rubulavirus” at several points throughout the text (e.g., lines 98, 687), particularly as we cannot distinguish whether the seropositivity we observe among the Pteropodidae is due to bat pararubula- or orthorubulaviruses. (To clarify, although reactivity against MuV/PIV2 are a measure of seropositivity against the orthorubulaviruses, we do not necessarily interpret this as definitive evidence for the presence of an infection history of a specific genus. Our language now better reflects this technical distinction.)

2) Related to #1, the authors stated that “serological cross-reactivity was evaluated” on line 370, p. 18, while they also mentioned that “these results do not explicitly provide evidence of cross-reactivity” on lines 398-399, p. 19. These different statements are really confusing. If the authors could not evaluate the cross-reactivity due to the unrecorded vaccine history or infections, they may need to reconsider the aim of this experiment. Is it possible to declare at least vaccine history of these patients?

**REPLY**: Please also see comment above. Although the exact degree of cross-reactivity could not be explicitly determined, the co-reactivity data provide an upper bound for putative cross-reactivity. As stated in the text (lines 199–202): *“Specifically, reference control sera were defined as either having derived from patients after acute infection or vaccination with the virus of interest or having dominant titers (i.e., at least tenfold higher against the virus of interest compared to related viruses).”*

3) Related to #1, the numbers of control sera used for the evaluation of the cross-reactivity of antigens seem different among the viruses. In example, while 23 WNV-infected sera have been incorporated for this experiment (l. 201, p. 10), none of them were used for WNV (NA in Table S2) and only 11 were listed for DENV2. The authors may want to explain how they chosen the sera used for each assay.

**REPLY**: All control sera were defined as being seropositive against the specified virus. We now state (lines 411–413): *“Due to limited availability, sometimes only a subset of the human reference sera could be used and the exact number of serum samples tested for each virus is reported (S2 Table).”*

4) “suggesting that the wild bats examined in this study had predominantly seroconverted against animal viruses, rather than directly against human pathogens” (l. 607-608, p. 33) should be over discussion. This is just one of the speculations to explain why serum reacted with antigens without neutralization activity, and the authors did not prove it in the present study. Better to omit from the manuscript.

**REPLY:** We thank the reviewer for raising this point and acknowledge that neutralization tests with animal viruses (which we do not have access to) would be necessary to explicitly demonstrate that the antibodies in this study were specific to animal-adapted, rather than human-adapted, viruses. Nevertheless, because it is already known that bat orthoflavi-, orthorubula-, and pararubulaviruses are circulating among some of the species examined in this study, we still consider it highly probable that bat-adapted, rather than human-adapted, viruses are responsible for the seropositivity we observe. We think including this interpretation in the Discussion is still valuable for readers who might otherwise incorrectly conclude that we are providing specific evidence for the circulation of human-adapted viruses in animals.

To address the reviewer’s point, we have now qualified our statement as follows (lines 643–650): “*Only 1/10 mIIFT-positive sera were reactive in virus-specific neutralization tests (Table 2), suggesting that the antibodies detected by mIIFT lacked substantial neutralizing activity against the respective viruses. This is plausibly due to seroconversion against related animal viruses (e.g., bat orthoflaviviruses, orthorubula-, and pararubulaviruses), rather than against the human viruses that were tested. However, to explicitly demonstrate that the antibodies in this study were specific to animal-adapted, rather than human-adapted, viruses, neutralization tests would need to be performed with both human and animal viruses in parallel.”*

5) I could not find the corresponding results in Fig. 2a for “despite the relatively high seroprevalence of flaviviruses in African bats, no seropositive samples against flaviviruses were found in European Vespertilionidae (Fig. 2a)”. Please update the figure based on the results the authors would like to refer.

**REPLY**: We have altered the sentence by mentioning the respective bat species/genera to make it more clear to which genera in Fig. 2a we are referring. It now reads (line 653–656):

*“For example, despite the relatively high seroprevalence of flaviviruses in African Pteropodidae (e.g., Eidolon, Rousettus), no seropositive samples against flaviviruses were found in European Vespertilionidae (e.g., Myotis, Nyctalus) (Fig. 2a), emphasizing that RNA virus distribution and concomitant risk to humans is highly dependent on local ecological factors.”*

6) This reviewer is interested the images of each mIIFT assay. Instead of performing neutralization assays for all viruses tested positive, the authors may be able to check their images for the signal localizations. I assume if the signals distribute widely throughout the cells, it may suggest the serum might react with envelope proteins as well as cytoplasmic ones, to partly prove the more specific reactivity to the virus tested.

**REPLY**: Although no quantitative signal localization was performed, qualitative observations about bright, localized fluorescent signal in some cells (as opposed to diffuse background signal in all cells) helped inform the mIIFT scoring. Clustering of positive signal around foci of infection (e.g., see S5 Fig.) contributed to positive signal designation. Representative images have been provided (Fig. S3, S4, S5), but given the large size of our dataset (n=1,589 and 14 different viral antigens), it was not logistically feasible to take and store images for every sample. Instead, two researchers looked at the mIIFT signals independently and documented the data in a lab book.
