# Supplementary material for: A multiregional serosurvey of RNA virus-reactive antibodies in bats and rodents reveals the enzootic presence of flavi- and paramyxoviruses in African Pteropodidae
Source: PLoS Negl Trop Dis. 2026 Oct 9;20(10):e0013689. doi: 10.1371/journal.pntd.0013689 (PMC13674865; doi:10.1371/journal.pntd.0013689)
Supplement: Peer review history

# Peer review: Decision Letter 0

**DOI:** 10.1371/journal.pntd.0013689.r001

**Authors:** Abdallah Samy

19 Jan 2026

A global serosurvey of RNA virus-reactive antibodies in bats and rodents reveals the enzootic presence of flavi- and paramyxoviruses in African Pteropodidae

PLOS Neglected Tropical Diseases

Dear Dr. Müller,

Thank you for submitting your manuscript to PLOS Neglected Tropical Diseases. After careful consideration, we feel that it has merit but does not fully meet PLOS Neglected Tropical Diseases's publication criteria as it currently stands. Therefore, we invite you to submit a revised version of the manuscript that addresses the points raised during the review process.

Please submit your revised manuscript within by Mar 20 2026 11:59PM. If you will need more time than this to complete your revisions, please reply to this message or contact the journal office at plosntds@plos.org. When you're ready to submit your revision, log on to https://www.editorialmanager.com/pntd/ and select the 'Submissions Needing Revision' folder to locate your manuscript file.

Please include the following items when submitting your revised manuscript:

* A letter that responds to each point raised by the editor and reviewer(s). You should upload this letter as a separate file labeled 'Response to Reviewers'. This file does not need to include responses to any formatting updates and technical items listed in the 'Journal Requirements' section below.

* A marked-up copy of your manuscript that highlights changes made to the original version. You should upload this as a separate file labeled 'Revised Manuscript with Track Changes'.

* An unmarked version of your revised paper without tracked changes. You should upload this as a separate file labeled 'Manuscript'.

If you would like to make changes to your financial disclosure, competing interests statement, or data availability statement, please make these updates within the submission form at the time of resubmission. Guidelines for resubmitting your figure files are available below the reviewer comments at the end of this letter.

We look forward to receiving your revised manuscript.

Kind regards,

Abdallah Samy

Section Editor

PLOS Neglected Tropical Diseases

Shaden Kamhawi

co-Editor-in-Chief

PLOS Neglected Tropical Diseases

orcid.org/0000-0003-4304-636XX

Paul Brindley

co-Editor-in-Chief

PLOS Neglected Tropical Diseases

orcid.org/0000-0003-1765-0002

**Additional Editor Comments:**

Please give careful consideration to all reviewer comments and ensure that each concern is thoroughly addressed prior to the submission of a revised version of your manuscript.

**Journal Requirements:**

If the reviewer comments include a recommendation to cite specific previously published works, please review and evaluate these publications to determine whether they are relevant and should be cited. There is no requirement to cite these works unless the editor has indicated otherwise.

1) We ask that a manuscript source file is provided at Revision. Please upload your manuscript file as a .doc, .docx, .rtf or .tex. If you are providing a .tex file, please upload it under the item type u2018LaTeX Source Fileu2019 and leave your .pdf version as the item type u2018Manuscriptu2019.

2) Please insert an Ethics Statement at the beginning of your Methods section, under a subheading 'Ethics Statement'. It must include:

i) The full name(s) of the Institutional Review Board(s) or Ethics Committee(s)

ii) The approval number(s), or a statement that approval was granted by the named board(s).

3) Please upload all main figures as separate Figure files in .tif or .eps format. For more information about how to convert and format your figure files please see our guidelines:

https://journals.plos.org/plosntds/s/figures

4) Some material included in your submission may be copyrighted. According to PLOSu2019s copyright policy, authors who use figures or other material (e.g., graphics, clipart, maps) from another author or copyright holder must demonstrate or obtain permission to publish this material under the Creative Commons Attribution 4.0 International (CC BY 4.0) License used by PLOS journals. Please closely review the details of PLOSu2019s copyright requirements here: PLOS Licenses and Copyright. If you need to request permissions from a copyright holder, you may use PLOS's Copyright Content Permission form.

Please respond directly to this email and provide any known details concerning your material's license terms and permissions required for reuse, even if you have not yet obtained copyright permissions or are unsure of your material's copyright compatibility. Once you have responded and addressed all other outstanding technical requirements, you may resubmit your manuscript within Editorial Manager.

Potential Copyright Issues:

i) Figure 1. Please (a) provide a direct link to the base layer of the map (i.e., the country or region border shape) and ensure this is also included in the figure legend; and (b) provide a link to the terms of use / license information for the base layer image or shapefile. We cannot publish proprietary or copyrighted maps (e.g. Google Maps, Mapquest) and the terms of use for your map base layer must be compatible with our CC BY 4.0 license.

Note: if you created the map in a software program like R or ArcGIS, please locate and indicate the source of the basemap shapefile onto which data has been plotted.

If your map was obtained from a copyrighted source please amend the figure so that the base map used is from an openly available source. Alternatively, please provide explicit written permission from the copyright holder granting you the right to publish the material under our CC BY 4.0 license.

If you are unsure whether you can use a map or not, please do reach out and we will be able to help you. The following websites are good examples of where you can source open access or public domain maps:

* U.S. Geological Survey (USGS) - All maps are in the public domain. (http://www.usgs.gov)

* PlaniGlobe - All maps are published under a Creative Commons license so please cite u201cPlaniGlobe, http://www.planiglobe.com, CC BY 2.0u201d in the image credit after the caption. (http://www.planiglobe.com/?lang=enl)

* Natural Earth - All maps are public domain. (http://www.naturalearthdata.com/about/terms-of-use/).

**Reviewers' Comments:**

Reviewer's Responses to Questions

**Key Review Criteria Required for Acceptance?**

As you describe the new analyses required for acceptance, please consider the following:

**Methods**

-Are the objectives of the study clearly articulated with a clear testable hypothesis stated?

-Is the study design appropriate to address the stated objectives?

-Is the population clearly described and appropriate for the hypothesis being tested?

-Is the sample size sufficient to ensure adequate power to address the hypothesis being tested?

-Were correct statistical analysis used to support conclusions?

-Are there concerns about ethical or regulatory requirements being met?

Reviewer #1: The methods need to be explained in more detail in some areas:

Line 187: which surgical procedures are the authors referring to in the frame of sampling? Drawing blood from a vein or artery would not be considered a surgical procedure, correct?

Line 211: why were all OD values multiplied by 1,000? This step should be explained.

Lines 234 – 248: why were STNs for YFV and MuV virus selected from all analyzed viruses? This should be explained for a better transparency.

Line 295: “if no information was available, a value of 12 years was assigned”. How was that number selected? This needs to be explained.

Reviewer #2: See combined comments below

**********

**Results**

-Does the analysis presented match the analysis plan?

-Are the results clearly and completely presented?

-Are the figures (Tables, Images) of sufficient quality for clarity?

Reviewer #1: The figures and tables are clear and of good quality.

Regarding the presentation of the results, I have the following suggestions:

Lines 367-373: I am not completely following this approach. If a bat species does not bind the anti-bat IgG conjugate equally well as others, but is still considered positive in the mIIFT, how are the results evaluated then? A quantitative interpretation is precluded from this, but how is an unspecific reaction excluded? This whole approach needs to be explained in a more comprehensive matter.

Table 1: as mentioned above, I question the approach of comparing the seroprevalence in species that are only analyzed in one target region (e.g. Eidolon helvum and Rousettus aegyptiacus). For some other bat and rodent species (Epomops franqueti, Hypsignatus monstrosus, Micropterus puillus, Apodemus sylvaticus, Rattus norvegicus, Microtus arvalis, Clethrionomys glareolus) there are specimen from two countries available, but at low, sometimes simple-digit sample numbers. In my view, the validity of this approach needs at least a better explanation.

Line 389-390: “sera with high background signal or inconclusive results were rated negative”. Why were these samples not excluded from the study? Rating them negative will affect the overall result.

Lines 394 – 398: only one out of 20 tested mIIFT positive sera was reactive in the virus-specific SNT, which is here explained by a possible cross-reactivity with a related virus. The possibility of false positive mIIFT results needs to be discussed as well, and moved into the discussion section. I would also suggest to test the sera that are positive for YFV and DENV2 in a DENV2 specific neutralization assay.

Line 401 – 404: again, how is a false positive result excluded here? > 45% of sera reacting with two or more virus families is a high number. How was this assured?

Line 419 – 426: see above, not sure of analyzing data from species only samples in one zone are sufficiently relevant. For Rattus, samples from both zones were compared, but at relatively low numbers.

Lines 458 – 460: “comparisons about differences in seroprevalence between bats and rodents or between tropical and temperate climate zones are not explicitly supported by our data”, this means that the data argue against such a correlation? If so, this needs to be elaborated.

Reviewer #2: See combined comments below

**********

**Conclusions**

-Are the conclusions supported by the data presented?

-Are the limitations of analysis clearly described?

-Do the authors discuss how these data can be helpful to advance our understanding of the topic under study?

-Is public health relevance addressed?

Reviewer #1: I have some general concerns about the interpretation of these data. Theauthors raise some limitations, but they do not offer a sound solution. This should in my view be discussed more transparently.

My comments in detail:

Line 549 -555: here, authors raise the points that in my view hamper the interpretation of the data in the way it is done here, but they don’t correlate these concerns with their interpretation, which nonetheless in both cases needs to be done.

Lines 572 – 580: the possibility of a fecal-oral flavivirus transmission in bats is valid and I agree that this should be further explored in relevant studies.

Lines 582 – 583: NiV is not a very good example for orthorubulavirus transmission.

Line 619: authors underline the high sensitivity of their approach, but how was the specificity (on virus family level) assured?

Line 633-634: the statement: “…makes this dataset unsuitable to broadly generalize about the relative seropositivity between …. tropical and temperate animals”, but this is the message of this study. Please consider rephrasing to make it more clear.

Lines 638 – 652: when discussing the limitations, the authors should also suggest means to confirm their findings, i.e. follow-up analysis with virus specific tests, more SNTs etc., even if this was technically impossible due to the low residual sample volumes in many cases. Also, are there serum and transudate samples available from the same animals in this study to compare the validity of serum and transudate?

Line 652: the logistic regression models are extensively explained in the methods and results section, but only briefly discussed. It is concluded in the last paragraph that the findings only indicate correlations, but cannot prove them. In this light, the manuscript title as well as the overall message of this manuscript seem to over-interpret the findings.

Reviewer #2: See combined comments below

**********

**Editorial and Data Presentation Modifications?**

Use this section for editorial suggestions as well as relatively minor modifications of existing data that would enhance clarity. If the only modifications needed are minor and/or editorial, you may wish to recommend “Minor Revision” or “Accept”.

Reviewer #1: (No Response)

Reviewer #2: See combined comments below

**********

**Summary and General Comments**

Use this section to provide overall comments, discuss strengths/weaknesses of the study, novelty, significance, general execution and scholarship. You may also include additional comments for the author, including concerns about dual publication, research ethics, or publication ethics. If requesting major revision, please articulate the new experiments that are needed.

Reviewer #1: This manuscript follows a serological approach to assess the prevalence of RNA viruses in bat and rodent species in different regions of the world. The multiplex approach by using the mIIFT is very suitable to address this. Although the scientific question behind the study is highly relevant, in my view the available data do not allow all conclusions drawn from it. My main concern is the comparability between frugivorous bats in tropical regions, and insectivorous bats in the Northern hemisphere. These are two fundamentally different scenarios with a whole number of variabilities, that I find it hard to point all observed differences to the bat or rodent species. In my view this is a considerable weakness of the study, and this point needs to be carefully addressed and discussed.

My comments in detail:

The title mentions a global serosurvey. In fact, samples from seven countries on four continents were analyzed, which is not a global survey.

Author summary:

Line 82/83: “we studied bats and rodents around the world” should be worded more carefully

Line 83: 14 viruses, add here: from 7 virus families

Introduction

Line 100: mention the goal / the benefit such studies would have.

Lines 108-111: the limitations referred to in general (low number of analyzed virus families / low number of sampled species per country) are also somewhat limiting the present study. This point should therefore be raised also in the discussion.

Lines 125-127: the authors then suggest a “systematic investigation of infection rates in bats and rodents”, which in my view needs to include sampling the same bat or rodent species at relevant numbers in different global regions, which was not pursued in the present study.

Line 141: exposure to filovirus antigen (instead of ‘with’)

Reviewer #2: This manuscript presents serological screening of bats and rodents across seven countries for antibodies to 14 human pathogenic RNA viruses. While the study addresses an important question about wildlife reservoir dynamics, several fundamental issues with study design, geographic scope, and analytical approach limit the validity and generalisability of the findings.

Major issues

- The use of "global" to describe this study is inappropriate and misleading. Seven countries, comprising two in Western Europe, three in West/Central Africa, one in SE Asia and one in Central America does not constitute global coverage. Major geographic regions including North America, South America, Australasia, and vast portions of Asia are completely unrepresented. The current terminology misrepresents the scope of the work and risks readers drawing conclusions about worldwide patterns that are not supported by the data. This is especially ths case given that only 1% of bat species and 0.5% of rodent species are sampled, and sampling is highly biased - 1015/1135 (89%) of bats were sampled in West/Central Africa, with the remaining in Germany (n=77 bats) and Panama (n=43 bats), and none in other sites. By comparison, rodents are sampeld in Western Europe (n=115 in Germany and n=165 in the Netherlands) and Thailand (n-174), with none in Africa or the Americas.

My concern with 'global' is easily fixed - it only appears in the title. Authors should remove "global" and frame alternatively as "multi-country" or "intercontinental". However, the generalisation of results from a small and biased sample set is more prevalent throughout - it is inappropriate to generalise findings within the abstract as applying to "tropical bats" when using such a narrow sample set, or by variables such as colony size, when there are so many potential confounders and too small a sample size to tease these confounders apart. The abstract and manuscript need a major reframing to avoid overstating the results. Additionally, results like Figure 2 must take into account sampling effort. It is meaningless to compare the number of viruses detected in genera from which one rodent (Microtus) or 14 individual bats (Coleura) were collected versus those where nearly 200 individual bats were sampled (Rousettus). A genus with one seropositive individual will show 100% of one viral family, while a genus with 200 sampled individuals has far more opportunity to detect multiple families. Analyses must account for differences in sample size before making such comparisons, or the figure adjusted so that the size of the circles represents sample size.

- Seasonality, reproductive status or age of individuals sampled is not addressed in the methods, despite it being well documented as a determinant of serostatus and included in the modelling later on. The effect of these covariates have also been shown to vary by viral family and host species. The absence of recognition of this within the study design, manuscript, and provision of data is a major gap.

- The choice of viruses included in the panels are not sufficiently justified. I can understand why most of the human viruses were included, but the rationale for excluding viral genera that are known to have strong associations with the bat and rodent reservoirs being screened is unclear (e.g. the huge diversity of paramyxoviruses that this group and others have demonstrated in African bats - but are only distantly related to measles and mumps). This includes viruses (or very close relatives of viruses) that are known to have transmitted to humans and for which control sera would have been available (e.g. pararubulavviruses and henipaviruses).

- The methods do not describe how seropositivity was determined for each assay (I see it is later presented in the results instead) and the reason for the use of VNTs for YFV was not described.

- The abstract implies that tropical vs temperate bats were statistically compared, but this is not the case. Similarly, it is not clear in the abstract that no statistical analyses were performed on rodent results. The authors restrict analyses to the tropical bat data for further analyses because it has high seroprevalence, removing the opportunity of comparing with low-prevalence bats. Using regional differences in sample size as an argument for this approach serves to highlight the weakness of the study design in the first place. The authors admit that "many bat families were only sampled in one region" and that collinearity and confounding are substantial problems, but proceed with the subsetted analyses anyway. Saying "This should be borne in mind when interpreting" is insufficient when the confounding is this severe.

Lifespan data are notoriously unreliable and inconsistently determined. Here, where there is missing data for a particular species the authors borrow data from related species, introducing an additional layer of uncertainty. Then, arbitrary assignment of 12 years when no data exist also introduces substantial uncertainty - all of which propagate through the analyses (leading to convergence issues later on).

- I have not commented in detail on the results or discussion, as I feel the issues highlighted above with the methods (e.g. providing sufficient detail/raw data on the age and reproductive status of individuals sampled and the seasonality of sampling) preclude interpretation. The statistical models show substantial uncertainty (wide credible intervals) that undermine the conclusions about risk factors

- The github link was broken

**********

PLOS authors have the option to publish the peer review history of their article (what does this mean?). If published, this will include your full peer review and any attached files.

If you choose “no”, your identity will remain anonymous but your review may still be made public.

**Do you want your identity to be public for this peer review?**  For information about this choice, including consent withdrawal, please see our Privacy Policy.

Reviewer #1: No

Reviewer #2: No

**Figure resubmission:**

While revising your submission, we strongly recommend that you use PLOS’s NAAS tool (https://ngplosjournals.pagemajik.ai/artanalysis) to test your figure files. NAAS can convert your figure files to the TIFF file type and meet basic requirements (such as print size, resolution), or provide you with a report on issues that do not meet our requirements and that NAAS cannot fix.

**Reproducibility:**

To enhance the reproducibility of your results, we recommend that authors of applicable studies deposit laboratory protocols in protocols.io, where a protocol can be assigned its own identifier (DOI) such that it can be cited independently in the future. Additionally, PLOS ONE offers an option to publish peer-reviewed clinical study protocols. Read more information on sharing protocols at https://plos.org/protocols?utm_medium=editorial-email&utm_source=authorletters&utm_campaign=protocols

# Author comment: Author response to Decision Letter 1

**DOI:** 10.1371/journal.pntd.0013689.r002

21 Mar 2026

Attachment Submitted filename: Response-to-reviewer-comments.Emanuel et al.pdf

# Peer review: Decision Letter 1

**DOI:** 10.1371/journal.pntd.0013689.r003

**Authors:** David Safronetz

20 Jul 2026

PNTD-D-25-01917R1

A multiregional serosurvey of RNA virus-reactive antibodies in bats and rodents reveals the enzootic presence of flavi- and paramyxoviruses in African Pteropodidae

PLOS Neglected Tropical Diseases

Dear Dr. Müller,

Thank you for submitting your manuscript to PLOS Neglected Tropical Diseases. After careful consideration, we feel that it has merit but does not fully meet PLOS Neglected Tropical Diseases's publication criteria as it currently stands. Therefore, we invite you to submit a revised version of the manuscript that addresses the points raised during the review process.

Please submit your revised manuscript within by Aug 19 2026 11:59PM. If you will need more time than this to complete your revisions, please reply to this message or contact the journal office at plosntds@plos.org. When you're ready to submit your revision, log on to https://www.editorialmanager.com/pntd/ and select the 'Submissions Needing Revision' folder to locate your manuscript file.

Please include the following items when submitting your revised manuscript:

* A letter that responds to each point raised by the editor and reviewer(s). You should upload this letter as a separate file labeled 'Response to Reviewers'. This file does not need to include responses to any formatting updates and technical items listed in the 'Journal Requirements' section below.

* A marked-up copy of your manuscript that highlights changes made to the original version. You should upload this as a separate file labeled 'Revised Manuscript with Track Changes'.

* An unmarked version of your revised paper without tracked changes. You should upload this as a separate file labeled 'Manuscript'.

If you would like to make changes to your financial disclosure, competing interests statement, or data availability statement, please make these updates within the submission form at the time of resubmission. Guidelines for resubmitting your figure files are available below the reviewer comments at the end of this letter.

As the corresponding author, your ORCID iD is verified in the submission system and will appear in the published article. PLOS supports the use of ORCID, and we encourage all coauthors to register for an ORCID iD and use it as well. Please encourage your coauthors to verify their ORCID iD within the submission system before final acceptance, as unverified ORCID iDs will not appear in the published article. Only the individual author can complete the verification step; PLOS staff cannot verify ORCID iDs on behalf of authors.

We look forward to receiving your revised manuscript.

Kind regards,

David Safronetz, Ph.D.

Section Editor

PLOS Neglected Tropical Diseases

David Safronetz

Section Editor

PLOS Neglected Tropical Diseases

Shaden Kamhawi

co-Editor-in-Chief

PLOS Neglected Tropical Diseases

orcid.org/0000-0003-4304-636XX

Paul Brindley

co-Editor-in-Chief

PLOS Neglected Tropical Diseases

orcid.org/0000-0003-1765-0002

**Additional Editor Comments (if provided):**

**Reviewers' Comments:**

Reviewer's Responses to Questions

**Key Review Criteria Required for Acceptance?**

As you describe the new analyses required for acceptance, please consider the following:

**Methods**

-Are the objectives of the study clearly articulated with a clear testable hypothesis stated?

-Is the study design appropriate to address the stated objectives?

-Is the population clearly described and appropriate for the hypothesis being tested?

-Is the sample size sufficient to ensure adequate power to address the hypothesis being tested?

-Were correct statistical analysis used to support conclusions?

-Are there concerns about ethical or regulatory requirements being met?

Reviewer #3: The methods are generally well described and appropriate for a broad exploratory serosurvey. The study design is retrospective and heterogeneous, but this is clearly acknowledged by the authors. The sampled population is described in sufficient detail and is suitable for descriptive surveillance and hypothesis generation, although not for strong general comparisons across host groups, regions, or climate zones. The overall sample size is impressive, but the effective sample size for some statistical comparisons is limited by uneven sampling across species, regions, years, and ecological traits. The statistical analysis is carefully presented and the use of multivariable Bayesian logistic regression is reasonable for exploring associations within the tropical bat subset. However, because many predictors are inferred at the species level and several variables are strongly confounded, the models should be interpreted strictly as hypothesis-generating and not as identifying independent causal risk factors. I have no major concerns regarding ethical or regulatory requirements, as the manuscript provides an extensive ethics statement and lists the relevant permits and approvals.

Reviewer #4: See "Summary and General Comments" section.

**********

**Results**

-Does the analysis presented match the analysis plan?

-Are the results clearly and completely presented?

-Are the figures (Tables, Images) of sufficient quality for clarity?

Reviewer #3: The results generally follow the analysis plan and are clearly presented. The descriptive serological findings are informative and supported by appropriate figures and supplementary material. The figures appear to be of sufficient quality and are generally clear. However, the presentation of the modelling results could be improved. At present, some important statistical information, particularly the posterior estimates and 94% credible intervals for the main predictors discussed biologically, is mainly available in supplementary figures. A concise main-text table summarizing the key model estimates, especially for colony size, frugivory/cave-dwelling, family, and region/year effects, would make the results easier to evaluate for non-specialist readers and would help distinguish robust associations from highly uncertain ones.

Reviewer #4: See "Summary and General Comments" section.

**********

**Conclusions**

-Are the conclusions supported by the data presented?

-Are the limitations of analysis clearly described?

-Do the authors discuss how these data can be helpful to advance our understanding of the topic under study?

-Is public health relevance addressed?

Reviewer #3: The conclusions are generally supported by the data, particularly regarding the value of broad serological surveillance and the evidence for widespread virus-reactive antibodies in the sampled wildlife populations. The authors also appropriately discuss how serology can complement direct virus detection and help guide future wildlife virus surveillance. The public health relevance is addressed in the context of reservoir hosts, spillover potential, and primary pandemic prevention. However, conclusions based on the statistical modelling should remain cautious. The limitations of the retrospective sampling design, uneven representation of host taxa and regions, and confounding among host traits are described, but some wording could still be softened to avoid implying that independent causal risk factors have been identified. I recommend consistently framing these modelling results as exploratory associations or hypothesis-generating findings rather than definitive predictors of infection risk.

Reviewer #4: See "Summary and General Comments" section.

**********

**Editorial and Data Presentation Modifications?**

Use this section for editorial suggestions as well as relatively minor modifications of existing data that would enhance clarity. If the only modifications needed are minor and/or editorial, you may wish to recommend “Minor Revision” or “Accept”.

Reviewer #3: (No Response)

Reviewer #4: (No Response)

**********

**Summary and General Comments**

Use this section to provide overall comments, discuss strengths/weaknesses of the study, novelty, significance, general execution and scholarship. You may also include additional comments for the author, including concerns about dual publication, research ethics, or publication ethics. If requesting major revision, please articulate the new experiments that are needed.

Reviewer #3: I would like to start by emphasizing that I find this manuscript valuable and scientifically interesting. The study is based on an impressive and unusually diverse sample set, including 1,135 bat and 454 rodent specimens collected from 63 sites across seven countries, and screened against 14 RNA viruses from seven virus families. I particularly appreciate the scale of the work and the effort required to assemble and analyse material from multiple host species, countries, and ecological settings. The multiplex immunofluorescence approach is well suited for such a broad exploratory serosurvey, especially given the known limitations of direct virus detection in field-collected wildlife samples. Overall, I consider this a useful contribution to wildlife virus surveillance. My comments below are intended mainly to help strengthen the interpretation of the data, particularly regarding the statistical analyses and the generalizability of the conclusions.

1. The statistical analysis is generally well described and represents a reasonable exploratory attempt to extract information from a heterogeneous retrospective dataset. It is more careful than the typical wildlife serosurvey regression, and the authors have clearly already tightened their causal language substantially in response to concerns about overgeneralization. However, the models should still be interpreted strictly as hypothesis-generating. Because many predictors are inferred at the species level while serostatus is measured at the individual level, and because species, family, geography, year, diet, colony size, and roosting ecology are strongly confounded, the models cannot robustly identify independent causal risk factors. I therefore recommend replacing “risk factor” throughout the abstract and discussion with more cautious wording, such as “associated factor”, “correlate”, or “predictor within this dataset”.

2. The presentation of the modelling results would benefit from a concise main-text table summarizing the posterior estimates and 94% credible intervals for the main predictors that are discussed biologically, especially colony size, frugivory/cave-dwelling, family, and region/year effects. At present, some important statistical evidence is buried in the supplementary material. Such a table would make it easier for non-statistical readers to assess which conclusions are well supported and which remain highly uncertain.

Reviewer #4: The present manuscript by J Emanuel et al. reported comprehensive serological surveillance of bats captured in various regions in the world. Since the study design mostly rely on immunofluorescence assays using virus-infected cells as antigens and only partly incorporate neutralization tests to validate the results, as the authors are aware, the present study must contain several limitations to interpret. However, the attempts to draw a broad overview of the serological presence of viral infections in bats, which are considered as the most important group of animals in the context of emerging infectious diseases, should be valuable for sharing with researchers in this field. I would like to raise some issues of the present manuscript below for improving the manuscript before further consideration, while I am not 100% sure that these comments are not against previous suggestions of reviewers.

1) The present study used 14 human pathogens as antigens. While the authors confirmed the reactivity of antisera for 17 pathogens to evaluate the assays, this reviewer has not been satisfied with this evaluation. Since the cross-reactivity can largely be explained by the amino-acid sequence identity between antigens, especially major antigens, such as nucleoproteins, matrix proteins, and envelope proteins, the authors may be able to show the potential coverage of their serological assays. In example, the cross-reactivity among flaviviruses may be dependent on the genetic complexes (doi: 10.3389/fimmu.2020.00334). The authors may be able to speculate the cross-reactivity using sequence comparison as well as literature review. Visualization of the coverages by either a figure or a table may be helpful for readers to understand how the results can be explained. Furthermore, I’m interested in whether bat viruses or bat-origin emerging viruses are included or not in the potentially cross-reactive viruses.

2) Related to #1, the authors stated that “serological cross-reactivity was evaluated” on line 370, p. 18, while they also mentioned that “these results do not explicitly provide evidence of cross-reactivity” on lines 398-399, p. 19. These different statements are really confusing. If the authors could not evaluate the cross-reactivity due to the unrecorded vaccine history or infections, they may need to reconsider the aim of this experiment. Is it possible to declare at least vaccine history of these patients?

3) Related to #1, the numbers of control sera used for the evaluation of the cross-reactivity of antigens seem different among the viruses. In example, while 23 WNV-infected sera have been incorporated for this experiment (l. 201, p. 10), none of them were used for WNV (NA in Table S2) and only 11 were listed for DENV2. The authors may want to explain how they chosen the sera used for each assay.

4) “suggesting that the wild bats examined in this study had predominantly seroconverted against animal viruses, rather than directly against human pathogens” (l. 607-608, p. 33) should be over discussion. This is just one of the speculations to explain why serum reacted with antigens without neutralization activity, and the authors did not prove it in the present study. Better to omit from the manuscript.

5) I could not find the corresponding results in Fig. 2a for “despite the relatively high seroprevalence of flaviviruses in African bats, no seropositive samples against flaviviruses were found in European Vespertilionidae (Fig. 2a)”. Please update the figure based on the results the authors would like to refer.

6) This reviewer is interested the images of each mIIFT assay. Instead of performing neutralization assays for all viruses tested positive, the authors may be able to check their images for the signal localizations. I assume if the signals distribute widely throughout the cells, it may suggest the serum might react with envelope proteins as well as cytoplasmic ones, to partly prove the more specific reactivity to the virus tested.

**********

PLOS authors have the option to publish the peer review history of their article (what does this mean?). If published, this will include your full peer review and any attached files.

If you choose “no”, your identity will remain anonymous but your review may still be made public.

**Do you want your identity to be public for this peer review?**  For information about this choice, including consent withdrawal, please see our Privacy Policy.

Reviewer #3: No

Reviewer #4: No

[NOTE: If reviewer comments were submitted as an attachment file, they will be attached to this email and accessible via the submission site. Please log into your account, locate the manuscript record, and check for the action link "View Attachments". If this link does not appear, there are no attachment files.]

**Figure resubmission:**

**Reproducibility:**

To enhance the reproducibility of your results, we recommend that authors of applicable studies deposit laboratory protocols in protocols.io, where a protocol can be assigned its own identifier (DOI) such that it can be cited independently in the future. Additionally, PLOS ONE offers an option to publish peer-reviewed clinical study protocols. Read more information on sharing protocols at https://plos.org/protocols?utm_medium=editorial-email&utm_source=authorletters&utm_campaign=protocols

# Author comment: Author response to Decision Letter 2

**DOI:** 10.1371/journal.pntd.0013689.r004

26 Aug 2026

Attachment Submitted filename: response_to_reviewers.docx

# Editor report: Decision Letter 2

**DOI:** 10.1371/journal.pntd.0013689.r005

**Authors:** Andrea Marzi

27 Sep 2026

Dear Dr. Müller,

We are pleased to inform you that your manuscript 'A multiregional serosurvey of RNA virus-reactive antibodies in bats and rodents reveals the enzootic presence of flavi- and paramyxoviruses in African Pteropodidae' has been provisionally accepted for publication in PLOS Neglected Tropical Diseases.

Before your manuscript can be formally accepted you will need to complete some formatting changes, which you will receive in a follow up email. A member of our team will be in touch with a set of requests.

Please note that your manuscript will not be scheduled for publication until you have made the required changes, so a swift response is appreciated.

IMPORTANT: The editorial review process is now complete. PLOS will only permit corrections to spelling, formatting or significant scientific errors from this point onwards. Requests for major changes, or any which affect the scientific understanding of your work, will cause delays to the publication date of your manuscript.

Should you, your institution's press office or the journal office choose to press release your paper, you will automatically be opted out of early publication. We ask that you notify us now if you or your institution is planning to press release the article. All press must be co-ordinated with PLOS.

Thank you again for supporting Open Access publishing; we are looking forward to publishing your work in PLOS Neglected Tropical Diseases.

Best regards,

Andrea Marzi

Section Editor

PLOS Neglected Tropical Diseases

David Safronetz

Section Editor

PLOS Neglected Tropical Diseases

Shaden Kamhawi

co-Editor-in-Chief

PLOS Neglected Tropical Diseases

orcid.org/0000-0003-4304-636XX

Paul Brindley

co-Editor-in-Chief

PLOS Neglected Tropical Diseases

orcid.org/0000-0003-1765-0002

***********************************************************

p.p1 {margin: 0.0px 0.0px 0.0px 0.0px; line-height: 16.0px; font: 14.0px Arial; color: #323333; -webkit-text-stroke: #323333}span.s1 {font-kerning: none

# Editor report: Acceptance letter

**DOI:** 10.1371/journal.pntd.0013689.r006

**Authors:** Andrea Marzi

Dear Dr. Müller,

We are delighted to inform you that your manuscript, "A multiregional serosurvey of RNA virus-reactive antibodies in bats and rodents reveals the enzootic presence of flavi- and paramyxoviruses in African Pteropodidae," has been formally accepted for publication in PLOS Neglected Tropical Diseases.

We have now passed your article onto the PLOS Production Department who will complete the rest of the publication process. All authors will receive a confirmation email upon publication.

The corresponding author will soon be receiving a typeset proof for review, to ensure errors have not been introduced during production. Please review the PDF proof of your manuscript carefully, as this is the last chance to correct any scientific or type-setting errors. Please note that major changes, or those which affect the scientific understanding of the work, will likely cause delays to the publication date of your manuscript. Note: Proofs for Front Matter articles (Editorial, Viewpoint, Symposium, Review, etc...) are generated on a different schedule and may not be made available as quickly.

Soon after your final files are uploaded, the early version of your manuscript will be published online unless you opted out of this process. The date of the early version will be your article's publication date. The final article will be published to the same URL, and all versions of the paper will be accessible to readers.

For Research Articles, you will receive an invoice from PLOS for your publication fee after your manuscript has reached the completed accept phase. If you receive an email requesting payment before acceptance or for any other service, this may be a phishing scheme. Learn how to identify phishing emails and protect your accounts at https://explore.plos.org/phishing.

Thank you again for supporting open-access publishing; we are looking forward to publishing your work in PLOS Neglected Tropical Diseases.

Best regards,

Shaden Kamhawi

co-Editor-in-Chief

PLOS Neglected Tropical Diseases

Paul Brindley

co-Editor-in-Chief

PLOS Neglected Tropical Diseases
